# Is Obesity a Modifiable Risk Factor in Multiple Sclerosis? Mechanistic Insights into Neuroinflammation and Oxidative Damage

**DOI:** 10.3390/pathophysiology33010005

**Published:** 2026-01-13

**Authors:** Fani-Niki Varra, Olga Pagonopoulou, Michail Varras, Viktoria-Konstantina Varra, Panagiotis Theodosis-Nobelos

**Affiliations:** 1 Department of Pharmacy, School of Health Sciences, Frederick University, Nicosia 1036, Cyprus; fannyvarra@gmail.com (F.-N.V.); hsc.np@frederick.ac.cy (P.T.-N.); 2 Medical School, Democritus University of Thrace, 68100 Alexandroupolis, Greece; 3 Laboratory of Neurophysiology, Medical School, Democritus University of Thrace, 68100 Alexandroupolis, Greece; opagonop@med.duth.gr; 4 Fourth Department of Obstetrics and Gynecology, ‘Elena Venizelou’ General and Maternity Hospital, 11521 Athens, Greece; 5 Department of Pharmacy, School of Health Sciences, National and Kapodistrian University of Athens, Panepistimiopolis of Zographou, Zographou, 15771 Athens, Greece; nadv.ars2016@gmail.com; 6 Division of Aesthetics and Cosmetic Science, Department of Biomedical Sciences, School of Health and Welfare Sciences, University of West Attica, Aigaleo, 12243 Athens, Greece

**Keywords:** obesity, multiple sclerosis, oxidative stress, inflammation, cytokines, adipokines, antioxidants, natural antioxidants

## Abstract

**Introduction**: Multiple sclerosis (MS) is a chronic autoimmune inflammatory disorder of the central nervous system (CNS) that leads to demyelination of CNS neurons and is influenced by genetic, environmental, and lifestyle factors, including diet and obesity. **Methods**: This review aims to analyze at the molecular level the relationship between obesity, as a chronic inflammatory condition, and the pathophysiology of MS, as a chronic autoimmune inflammatory disease, in order to understand the complex links between obesity and MS through a search of the PubMed and Google Scholar databases. **Discussion**: Chronic inflammation and OS are interconnected processes, causing a toxic state, which contributes to the development of CNS neuroinflammation and neuronal damage, resulting in neuronal demyelination and the onset of MS. Adipose tissue is a complex endocrine organ; in addition to being a lipid storage organ, it secretes cytokines and adipokines, which are involved in the regulation of hormones, metabolism, inflammation, and whole-body homeostasis. Obesity triggers chronic low-grade inflammation, disruption of the blood–brain barrier (BBB) and brain metabolism, infiltration of the CNS by immune cells, production of ROS, and generation of oxidative stress (OS). Anti-inflammatory and pro-inflammatory adipokines are also implicated in MS and obesity. **Conclusions**: Obesity affects MS through common underlying mechanisms and seems to be a modifiable risk factor. Antioxidant and anti-inflammatory compounds with multi-functional characteristics could be additional tools to slow the progression of MS and its promotion through obesity while also offering potential treatment options for both conditions via their multi-targeting characteristics.

## 1. Introduction

Obesity is characterized by an abnormal or excessive accumulation of body fat associated with adverse health outcomes [1]. It reflects a chronic, low-grade inflammatory state linked to a wide range of pathological conditions, including MetS and IR, and various diseases such as T2DM, elevated BP, CVDs, NAFLD, kidney and musculoskeletal disorders, infertility, psychological disorders, certain cancers, and autoimmune diseases [2,3,4,5,6,7]. Dietary shifts in recent years, marked by increased intake of high-fat and high-sugar foods, have contributed significantly to the global obesity epidemic [4,8]. As reported by the WHO, an estimated 59% of the global population falls into the overweight (BMI: 25–30 kg/m^2^) or obese (BMI > 30 kg/m^2^) categories [9].

MS is a chronic, autoimmune, inflammatory disease of the CNS, involving demyelination—the loss of the myelin sheath surrounding nerve fibers—and neurodegeneration, which disrupts or blocks the transmission of nerve impulses along axons [10]. MS can be clinically categorized into three forms: (a) RRMS, (b) SPMS, and (c) PPMS [11]. The disease mainly affects young adults, with onset most commonly between 20 and 50 years of age and an average age at onset of approximately 30 years [12]. However, MS can also develop in childhood or after the age of 60 [10], with its global prevalence steadily rising in recent decades, particularly among women [13,14,15,16]. 

MS is considered to arise from the complex interaction of genetic, environmental, and lifestyle factors, including diet and obesity [17,18,19]. Overweight and obese individuals are reported to face a markedly elevated risk of developing MS [20]. Obesity at the time of diagnosis in women with MS has been linked to a relapsing disease course [21]. Moreover, a positive correlation has been observed between obesity and disability severity in individuals with MS, as indicated by higher EDSS scores [22]. Additionally, comorbidities including T2DM, hypertension, hypercholesterolemia, and peripheral vascular disease appear to independently contribute to increased disability in individuals with MS [21]. Evidence also suggests that obesity during childhood, adolescence, and early adulthood is associated with an increased risk of developing MS [19,23]. Furthermore, obesity may influence both disease progression and treatment outcomes [10]. Notably, a correlation has been reported between the extent of disability and oxidative stress (OS) in MS patients [22].

The aim of the present study is to investigate at the molecular level the potential pathophysiological link between obesity, as a chronic inflammatory condition, and the pathogenesis of MS, which is likewise a chronic inflammatory autoimmune disease of the CNS. This includes the analysis of mechanisms such as the release of pro- and anti-inflammatory adipokines and cytokines, activation of inflammasomes, alterations in gut microbiota, and OS. Additionally, the present study examines the complementary role of multi-functional antioxidant compounds in the treatment of MS, particularly by mitigating chronic inflammation and OS, while also exploring the interrelation of these conditions from the perspective of treatment strategies.

## 2. Materials and Methods

A comprehensive literature search was conducted to identify studies investigating pathophysiological and molecular mechanisms shared between obesity and multiple sclerosis (MS), as well as the effects of natural antioxidant compounds for MS. The databases PubMed and Google Scholar were searched, focusing primarily on publications from the past fifteen years to ensure inclusion of up-to-date evidence. Search terms combining keywords related to “obesity”, “multiple sclerosis”, “chronic inflammation”, “adipokines” (adiponectin, apelin, leptin, visfatin, resistin, plasminogen activator inhibitor-1 [PAI-1], chemerin, fatty acid-binding protein 4 [FABP-4]), “cytokines (TNF-α, IL-6, IL-8, IL-18, IL-1β, IL-10)”, “NLRP3 inflammasome” and “antioxidants (vitamin D, vitamin A, curcumin, resveratrol, quercetin) were used. Both original research and review articles were considered. Titles and abstracts were screened for relevance, and full texts of eligible articles were assessed to extract data relating to the proposed subsections: (i) pathophysiological associations between obesity and MS; (ii) the role of chronic inflammation; (iii) anti- and pro-inflammatory adipokines and cytokines; (iv) NLRP3 inflammasome activation; and (v) antioxidant compounds in the management of MS. Reference lists of the included articles were also examined to identify additional relevant studies.

## 3. Pathophysiological Association Between Obesity and Multiple Sclerosis

### 3.1. The Contribution of Chronic Inflammation to the Development of MS

Obesity and MS share common pathophysiological mechanisms that interconnect them, particularly through the promotion of chronic inflammation, alterations in adipokine and cytokine secretion, associations with oxidative stress, and dysregulation of both the inflammasome and the gut microbiota [24,25].

**Figure 1:**
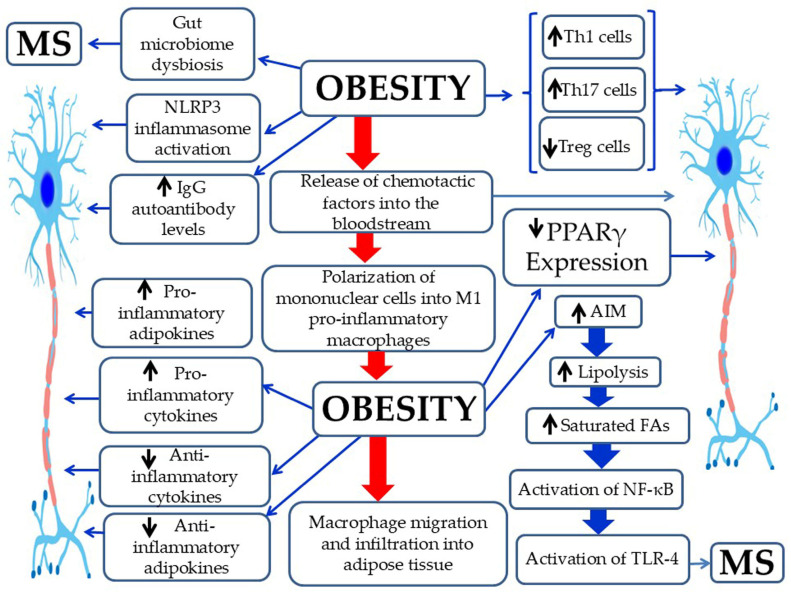
Schematic representation of the main mechanisms by which chronic inflammation contributes to the pathogenesis of multiple sclerosis in obesity.

Obesity is a metabolic condition characterized by adipocyte enlargement (hypertrophy) and proliferation (hyperplasia) [7,26]. This expansion of adipose tissue promotes the release of several chemotactic molecules into the bloodstream—including MCP-1, -2, -3, and -4, CXCL10, eotaxin, and CCL5/RANTES—thereby facilitating the recruitment of immune cells from the circulation [7] (Figure 1). These monocytes are subsequently activated into M1 pro-inflammatory macrophages, replacing the M2 anti-inflammatory phenotype typically present among those with a normal body weight range [7]. In adipose tissue, M1 macrophages release an increased amount of pro-inflammatory cytokines (such as IL-6, IL-1β, and TNF-α) and adipokines (such as leptin, visfatin, resistin, and PAI-1). Obesity leads to reduced secretion of anti-inflammatory adipokines (such as adiponectin and apelin) and cytokines (like IL-10) [7]. Overall, these adipose-tissue inflammatory shifts promote chronic, systemic, low-grade inflammation, a key factor in MetS, IR, T2DM, and other obesity-related diseases, including MS [7,27]. Obesity further increases pro-inflammatory Th1, Th17, and CD8+ T cells, as well as neutrophils [28], and reduces anti-inflammatory Th2 and Treg cells, needed for immune balance [29]. Furthermore, there is a decrease in iNKT cells and ILC2, along with diminished expression of PPAR-γ, a key regulator of adipose tissue homeostasis [30]. Chronic inflammation in adipose tissue leads to an altered adipokine profile marked by increased secretion of leptin, visfatin, resistin, and PAI-1 and decreased adiponectin, an adipokine with anti-inflammatory properties [7,27]. Similarly, early stages of MS are characterized by chronic inflammation within the CNS, which progresses to neurodegeneration in the later phases of the disease [31]. Chemokines, including MCP-1, -2, -3, and CCL5/RANTES, are key contributors in the pathophysiology of MS [32,33]. The immune profile seen in MS resembles that of obesity, characterized by elevated Th1 and Th17 cell levels and a concurrent reduction in Th2 and Treg lymphocytes [28]. The dominance of Th17 cells is particularly implicated in promoting autoimmune inflammation within the CNS, thereby contributing to MS pathogenesis [16]. In addition, increased concentrations of pro-inflammatory adipokines may exacerbate disease progression in obese individuals with MS [29]. The abnormal secretion of pro-inflammatory cytokines from adipocytes and macrophages in obesity is believed to contribute to MS development [16]. Moreover, excessive lipolysis in obesity, driven by AIM, in an attempt to counteract disease progression, leads to the secretion of significant quantities of SFAs [16]. These SFAs activate TLR-4, triggering CNS demyelination and inflammation through NF-κB signaling, thus promoting MS pathogenesis [16,34]. Finally, obesity is linked with enhanced generation of IgG autoantibodies that are implicated in the development of several autoimmune disorders, such as MS [16].

#### 3.1.1. Anti-Inflammatory Adipokines in MS

*Adiponectin*

**Figure 2:**
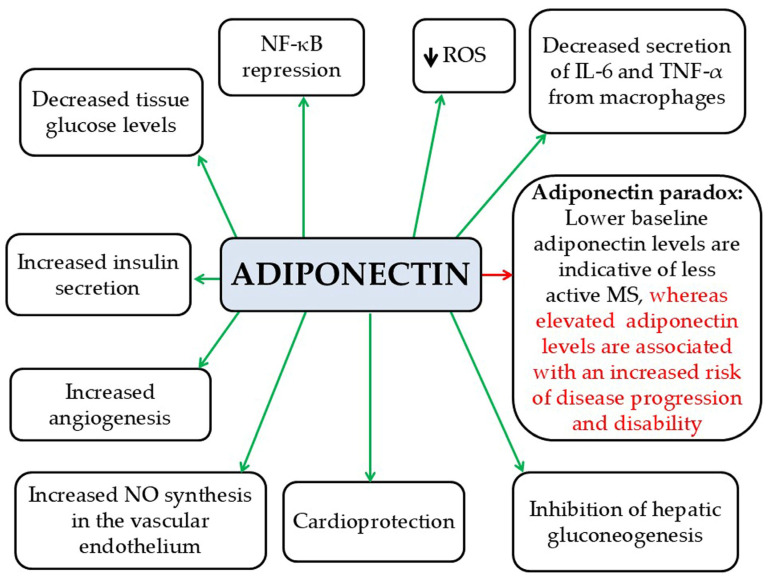
Protective actions of adiponectin in obesity and its controversial role in MS (negative effects on MS indicated in red color). Adiponectin serves as a biomarker for monitoring MS progression and severity.

Adiponectin is a key adipokine with anti-inflammatory effects. It is a 30 kDa peptide hormone predominantly secreted by white adipose tissue [7]. Its actions include (a) suppression of NF-κB activity, (b) reduction in TNF-α and IL-6 secretion from macrophages, (c) reduction of ROS production, (d) reduction of glucose levels in tissues, (e) increase in insulin secretion, (f) inhibition of hepatic gluconeogenesis, (g) cardioprotection, (h) increase in NO synthesis in the vascular endothelium, and (i) promotion of angiogenesis [7] (Figure 2). Serum adiponectin levels are reduced in individuals with obesity, IR, T2D, dyslipidemia, and CVDs [7]. Moreover, adiponectin synthesis is suppressed by pro-inflammatory cytokines such as TNF-α and IL-6 [7]. In a mouse model of EAE, adiponectin deficiency was shown to intensify lymphocyte activation and worsen disease severity [35]. Clinical studies demonstrated an “adiponectin paradox” in MS, where higher levels of adiponectin are associated with worse disease severity and prognosis, despite its expected anti-inflammatory and neuroprotective effects. Tehrani et al. found that female patients with RRMS exhibited higher adiponectin levels than healthy controls [36]. Similarly, Düzel et al. reported consistent findings [37]. Furthermore, elevated serum adiponectin levels at the time of MS diagnosis—before any treatment—have been linked to a greater risk of disease progression and disability [38]. Lower adiponectin levels have been observed in MS patients compared with healthy controls [39,40,41]. Therefore, adiponectin may be a valuable biomarker at the onset of MS, potentially assisting in predicting disease progression and severity [42]. The “adiponectin paradox” in MS requires further investigation, as adiponectin—typically anti-inflammatory—may exhibit pro-inflammatory effects in the context of MS. This “adiponectin paradox” highlights a complex phenomenon, suggesting that under certain conditions, such as those present in the MS environment, adiponectin may promote inflammatory immune responses instead of exerting its usual anti-inflammatory effects.

*Apelin*

**Figure 3:**
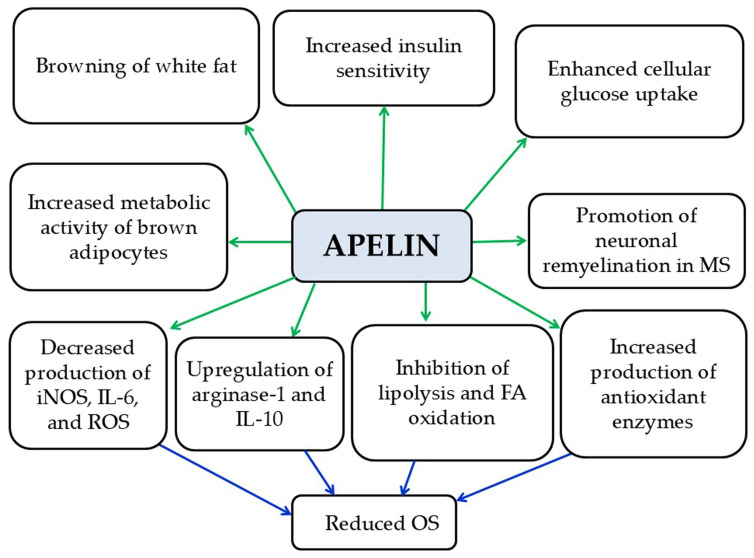
Protective actions of apelin in obesity and its potency towards obesity-associated MS (with blue arrows depicting the secondary positive effects of apelin on reducing OS).

Apelin is a small-molecular-weight peptide classified among the anti-inflammatory adipokines and is primarily secreted by adipocytes [7]. Its main actions include (a) promoting the differentiation and metabolic activity of brown adipocytes and inducing the browning of white adipose tissue; (b) increasing glucose uptake by cells; (c) enhancing insulin sensitivity; (d) inhibiting lipogenesis, lipolysis, and fatty acid oxidation; (e) promoting the synthesis of antioxidant enzymes; and (f) reducing OS [7] (Figure 3). Elevated serum apelin levels have been observed in individuals with obesity. This may be due either to peripheral apelin resistance or to a compensatory mechanism intended to counteract insulin resistance in peripheral tissues [7]. A preclinical study in N9 microglial cells demonstrated that apelin-13, the active form of apelin, can suppress LPS-induced production of iNOS and IL-6 while promoting an anti-inflammatory environment through the upregulation of anti-inflammatory markers such as IL-10 and arginase-1 [43]. Several clinical studies have reported conflicting findings regarding apelin levels in MS patients. Tehrani et al. reported decreased apelin levels in women with very early-stage RRMS, which showed a positive correlation with both EDSS scores and the number of relapses [36]. In contrast, Alpua et al. observed elevated apelin levels in RRMS patients compared to controls, although these levels did not correlate with disease severity or duration [44]. These discrepancies in clinical studies on apelin as a biomarker are likely attributable to variations in study design, patient populations, disease stages, and measurement methods, making it challenging to establish a consistent role for apelin in MS. Apelin and its receptor, APJ, are widely expressed in the CNS, particularly in neurons and oligodendrocytes [45]. However, preclinical studies indicate that apelin may promote remyelination in the context of MS. Specifically, Ito et al. suggested that APJ activation can enhance remyelination by modulating myelin-associated regulatory factors, particularly in demyelinating conditions in mouse models [46]. Furthermore, apelin seems to promote the differentiation of neural stem cells in an animal model of SCI [47]. These preclinical findings indicate that apelin may represent a potential therapeutic strategy for MS by supporting myelin repair, even while its role as a reliable biomarker in human studies remains unclear. 

#### 3.1.2. Pro-Inflammatory Adipokines in MS

*Leptin*

**Figure 4:**
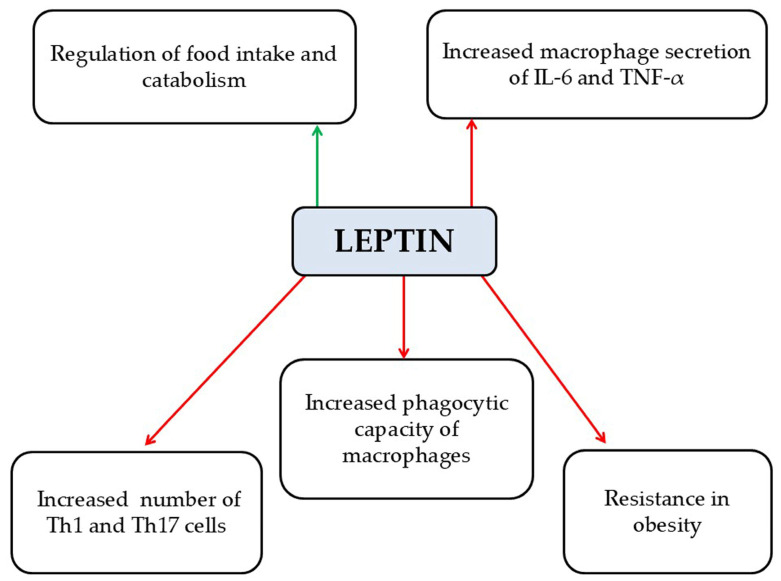
Actions of leptin related to the pathophysiology of multiple sclerosis (MS) (with red and green arrows depicting the negative and the positive effects, respectively).

Leptin is classified among the pro-inflammatory adipokines [48]. It is a peptide consisting of 167 amino acids with a molecular weight of 16 kDa [48]. Leptin is primarily secreted by white adipose tissue, and its circulating levels correlate directly with the mass of adipose tissue [49]. Its main functions include (a) control of appetite and metabolism through signaling to the CNS via specific receptors [48]; (b) upregulation of pro-inflammatory cytokines, including TNF-α and IL-6 [7]; (c) stimulation of pro-inflammatory Th1 and Th17 cell proliferation; and (d) promotion of OS [50,51] (Figure 4). In obesity, leptin resistance develops, leading to increased serum leptin levels [52], which may be linked to changes in the BBB observed in obese individuals [53]. Furthermore, increased leptin concentrations have been found in the CSF of patients with RRMS [54,55]. Ouyang et al. demonstrated that removal of leptin receptors reduced leukocyte infiltration into the CNS and alleviated BBB disruption in an EAE mouse model, suggesting that leptin receptor levels could potentially serve as a prognostic marker for disease progression [56]. However, it remains unclear whether CNS leptin receptor levels are primarily driven by peripheral inflammation and BBB penetration or by local production within the brain [56].

*Visfatin*

**Figure 5:**
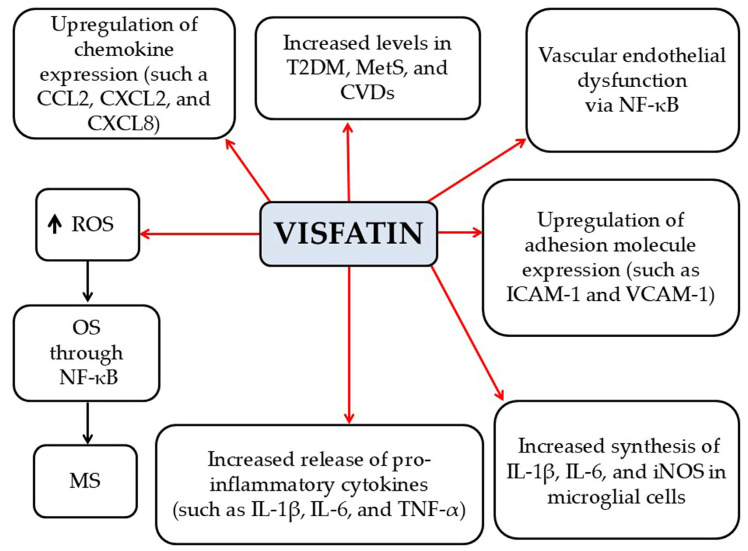
The negative effects of visfatin on chronic inflammation and oxidative stress associated with obesity and multiple sclerosis (MS).

Visfatin is a 52 kDa pro-inflammatory adipokine secreted by macrophages in visceral adipose tissue, bone marrow, skeletal muscles, liver, lungs, pancreas, heart, brain, and various other organs [7]. Visfatin upregulates the expression of chemokines CCL2, CXCL2, and CXCL8 (IL-8), as well as adhesion molecules such as ICAM-1 and VCAM-1, acting as a chemoattractant for monocytes and lymphocytes [57]. Moreover, it induces (a) secretion of pro-inflammatory cytokines such as IL-1β, IL-6, and TNF-α; (b) endothelial dysfunction via NF-κB signaling pathway; and (c) OS through NF-κB signaling [7] (Figure 5). Elevated visfatin levels have been reported in obesity, T2DM, MetS, and CVDs, while weight reduction in obese patients has been shown to decrease visfatin concentrations [7]. A preclinical study in microglial cells demonstrated that visfatin increases the synthesis of pro-inflammatory mediators, including IL-1β, IL-6, and iNOS in response to LPS stimulation [58]. This effect is associated with visfatin-induced activation of the inflammatory NF-κB pathway, leading to increased production of ROS and NO in these cells [58]. Visfatin levels have been reported to be increased in MS patients compared to healthy individuals [59], particularly in those with RRMS, and these levels show a positive correlation with TNF-α and a negative correlation with FoxP3 mRNA in T cells [60]. The elevated visfatin levels observed in MS patients suggest that visfatin may enhance neuroinflammatory processes within the CNS, thereby contributing to increased demyelination and progressive neurodegeneration.

*Resistin*

**Figure 6:**
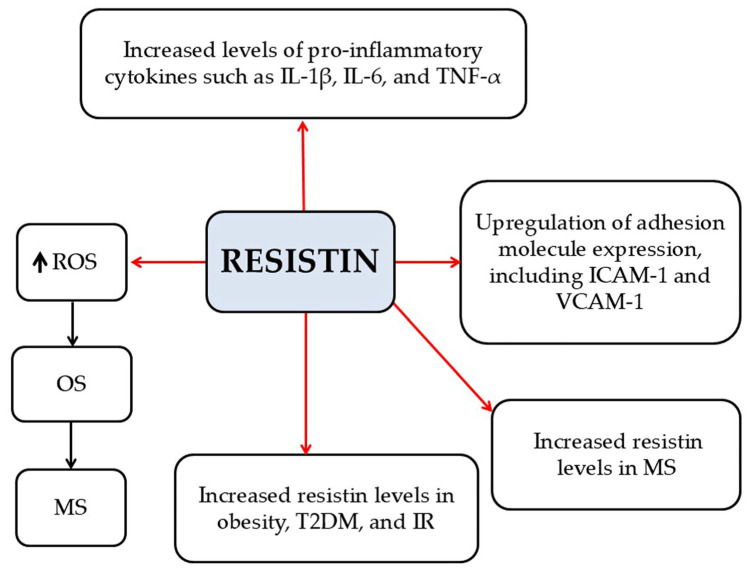
Promoting activities of resistin in chronic inflammation and OS associated with obesity and MS.

Resistin is a 12.5 kDa pro-inflammatory adipokine, mainly secreted by macrophages in adipose tissue [7]. Resistin (a) stimulates the production of pro-inflammatory cytokines, including IL-1β, IL-6, and TNF-α; (b) increases the expression of several adhesion molecules; and (c) enhances OS [7]. Some studies have reported that elevated serum resistin levels are associated with obesity, IR, and T2DM, while other studies have not confirmed these findings [7]. Moreover, Hossein-Nezhad et al. reported higher serum resistin levels in MS patients compared to controls, accompanied by increased IL-1β, TNF-α, and CRP levels [61]. In addition, in patients with RRMS, elevated serum resistin levels are correlated with decreased Treg activity, potentially contributing to a more severe disease progression [41] (Figure 6). Thus, resistin may adversely affect MS by acting as a pro-inflammatory cytokine that exacerbates disease progression. It promotes inflammation and autoimmune responses by activating inflammatory pathways, increasing the production of other pro-inflammatory cytokines, and recruiting inflammatory immune cells to the CNS. These effects can amplify neurodegeneration and demyelination, ultimately worsening MS pathology.

*PAI-1*

PAI-1 is a tPA [7,62,63]. Elevated activity of PAI-1 is linked to impaired fibrinolysis, leading to an increased risk of CVDs [7]. PAI-1 is produced by adipose tissue, fibroblasts, vascular endothelial cells, and immune cells, and its serum levels are elevated in obese individuals, showing correlations with IR, MetS, and atherosclerosis [7]. Elevated PAI-1 levels have also been observed in the serum and CSF of MS patients [64]. Furthermore, serum PAI-1 levels were higher in patients with active MS compared to those with stable disease and showed a positive association with neurological deterioration and disability, with the results not being definite and conclusive [64].

*Chemerin*

Chemerin is a pro-inflammatory adipokine involved in regulating adipocyte differentiation and is associated with obesity and MetS [65]. It functions as a chemoattractant for pDCs and macrophages activated through TLR-9 and HMGB1, thereby promoting type I interferon production [66]. Chemerin binds to the G protein-coupled receptors CMKLR1, GPR1, and CCRL2. The binding of chemerin to CCRL2 promotes macrophage infiltration and contributes to IR [67]. CMKLR1 is predominantly expressed on infiltrating lymphocytes, dendritic cells, and macrophages [68]. Studies on chemerin levels in MS have yielded conflicting results, possibly due to differences in patient populations, metabolic status, and research methods. Tomalka-Kochanowska et al. reported increased chemerin levels in MS patients, which are associated with obesity and higher body weight, reflecting chemerin’s strong link to adiposity and metabolic inflammation [69]. In contrast, Koskderelioglu et al. did not observe these associations, which may be attributed to differences in BMI distribution, disease stage, sample size, or the clinical characteristics of their study participants [70]. These discrepancies emphasize the multifactorial regulation of chemerin and highlight the necessity for further, larger, well-controlled clinical studies to elucidate its role in MS pathophysiology.

*FABP-4*

FABP-4 is a pro-inflammatory adipokine produced by adipocytes, monocytes, and macrophages [71]. FABP-4 expression is enhanced in response to TLR-2 stimulation [71]. FABP-4 levels are elevated in obese individuals compared to those who are overweight or of normal weight [72]. FABP-4 deficiency decreases the secretion of pro-inflammatory cytokines by suppressing the NF-κB pathway [73], whereas administration of recombinant FABP-4 promotes pro-inflammatory cytokine secretion through the p38/NF-κB pathway [74]. Moreover, FABP-4 is released from lipolysis of fat droplets into the bloodstream, reaching various organs, including the CNS [75,76]. Conversely, FABP-4 knockout mice exhibit reduced clinical severity in EAE, and their dendritic cells produce lower levels of pro-inflammatory cytokines [77,78]. Additionally, in pediatric MS patients, FABP-4 and leptin positively correlate with RRMS, suggesting that these adipokines may contribute to disease progression [74]. Furthermore, in adult MS patients, FABP-4 has been associated with greater disability independently of BMI [72]. Similarly, in women, higher serum FABP-4 levels correlate with elevated EDSS scores [72], whereas reduced miR-34a expression has been detected in PBMCs from patients with RRMS [79]. Taken together, these findings suggest that FABP-4 may serve as a prognostic marker for MS, especially in obese patients, due to its role at the intersection of metabolic regulation and neuroinflammation. Its involvement in adipokine networks, immune signaling, and lipid homeostasis further indicates that FABP-4 could contribute to both MS-related inflammation and neurodegeneration. Nevertheless, further experimental studies and well-designed clinical trials are required to elucidate the precise relevance of FABP-4 to MS pathophysiology.

#### 3.1.3. Pro-Inflammatory Cytokines in MS

*TNF-α*

**Figure 7:**
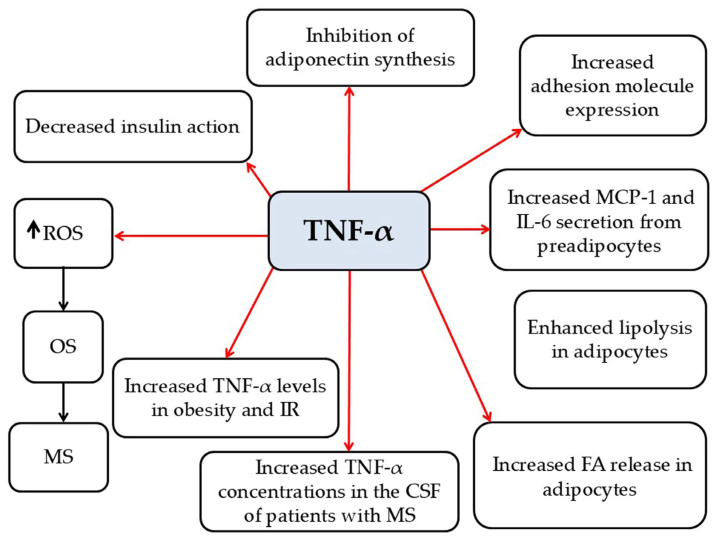
Harmful actions of TNF-α in chronic inflammation and OS, associated with obesity and MS.

TNF-α is a pro-inflammatory adipokine primarily generated by a wide range of cell types, including macrophages, T and B lymphocytes, adipocytes, vascular endothelial cells, astrocytes, neurons, and muscles [7,80,81]. Its biological actions are mediated through two receptors: TNF-R1 and TNF-R2 [7,81]. TNFR1 is ubiquitously expressed across virtually all cell types [82], while TNFR2 is predominantly localized to neurons, endothelial cells, and several immune cell populations [83]. Activation of TNFR1 is associated with pathogenic outcomes, while TNFR2 signaling is generally linked to protective effects [84]. In particular, soluble TNF-α predominantly signals via TNFR1, promoting processes such as cellular apoptosis, including oligodendrocytes [85], and driving chronic inflammation. In contrast, membrane-bound TNF-α primarily engages TNFR2, leading to the activation of genes involved in cell survival and resolution of inflammatory responses [81]. In conditions of obesity and IR, TNF-α expression is elevated, and its levels have been shown to decline following weight reduction [7]. Experimental studies indicate that TNF-α administration enhances IR in adipocytes [7]. Its actions include (a) stimulating MCP-1 and IL-6 secretion from preadipocytes [86]; (b) inhibiting adiponectin synthesis [87]; (c) promoting the release of FFAs from adipocytes [87]; (d) activating NF-κB, which increases adhesion molecule expression on endothelial and vascular smooth muscle cells, thereby contributing to atherogenesis [87]; (e) impairing insulin-mediated peripheral glucose uptake [7]; (f) enhancing lipolysis in adipocytes [7]; and (g) inducing ROS production, including superoxide anion radical, leading to OS [7] (Figure 7). TNF-α also appears to be involved in MS activation [88,89]. Sharief and Henges reported increased TNF-α levels in the CSF of patients with active MS [90], which correlated with disease severity and progression [90]. SNPs in the TNFR1 gene (TNFRSF1A), encoding TNFR1, have been associated with an increased risk of developing MS [91]. In EAE mouse models, TNF-α expression is upregulated, and exogenous TNF-α administration exacerbates disease progression [89,92,93]. Conversely, EAE mice lacking TNFR1 either show full resistance or develop milder disease [94,95]. Furthermore, TNFR2 deficiency aggravates disease severity [96,97,98]. Thus, TNF-α plays a pivotal role in MS, acting both as an inflammatory mediator and a neuroprotective factor through receptor-specific pathways. Its pro-inflammatory effects are primarily mediated by TNFR1, whereas TNFR2 activation supports remyelination and immune regulation. These opposing actions highlight the complexity of targeting TNF-α in MS and help explain the failure of non-selective TNF-α blockade in clinical trials. Future therapies will need to precisely modulate TNF-α signaling, inhibiting TNFR1-mediated detrimental effects while preserving or enhancing the neuroprotective functions of TNFR2.

*IL-6*

IL-6 is a pro-inflammatory cytokine that triggers the acute-phase inflammatory response [7,99,100]. It is produced by a variety of cell types, including adipocytes, endothelial cells, monocytes, T and B lymphocytes, fibroblasts, microglia, neurons, and pancreatic beta cells [7,99,100]. IL-6 signals either via its membrane-bound receptor IL-6Rα (classical signaling) or through the soluble receptor, sIL-6R [7,99]. Signaling through gp130 activates the JAK1/STAT3 pathway, promoting gene transcription [99], and also engages the MAPK pathway, leading to transcription of additional genes [99]. Serum IL-6 levels are elevated in obese individuals, as well as in patients with chronic inflammatory conditions and dyslipidemia, with adipose tissue contributing approximately one-third of circulating IL-6 [63,101]. The hypothalamus shows the highest expression of IL-6 receptors, suggesting a possible role for IL-6 in the regulation of appetite and food intake [63,101]. IL-6 also contributes to the differentiation of Th17 lymphocytes [99]. Notably, IL-6-deficient mice show resistance to EAE, although these findings are preliminary and require further investigation before definitive conclusions can be drawn [102,103]. Future studies are required to elucidate the precise mechanisms through which IL-6 contributes to MS progression and to develop strategies that selectively inhibit its pathogenic effects while preserving its essential physiological functions. Targeted therapies are particularly important, as IL-6 is also critical for normal immune responses and CNS homeostasis, and complete blockade could result in unintended adverse effects.

*IL-8*

IL-8 is a pro-inflammatory cytokine predominantly produced by monocytes and macrophages [101]. Oxidized LDL (ox-LDL) stimulates IL-8 production and release from macrophages derived from human atherosclerotic plaques and foam cells [101]. Additionally, IL-8 promotes the release of MMP-9 from neutrophils [104]. Elevated IL-8 levels have been found in obese individuals, as well as in patients with T2DM and MetS [105,106]. IL-8 contributes to BBB disruption and facilitates immune cell migration into the CNS, with MS patients exhibiting lower serum but higher CSF IL-8 levels compared to healthy controls [107]. Lund et al. demonstrated significantly higher serum IL-8 in untreated MS patients relative to controls, with levels decreasing following interferon-beta-1a therapy [108]. Similarly, Neuteboom et al. found that elevated IL-8 during pregnancy was associated with an increased risk of postpartum relapse [109].

*IL-18*

IL-18 is produced by both hematopoietic and non-hematopoietic cells, including monocytes, macrophages, keratinocytes, and mesenchymal cells [110]. A member of the IL-1 cytokine family, which consists of 11 cytokines that enhance innate immune responses, IL-18 plays a role in stimulating both innate and adaptive immunity [110]. It has also been implicated in the pathogenesis of MS and in the development of EAE [111]. Elevated IL-18 expression has been found in the serum and PBMCs of MS patients, as well as in the brain and spinal cord tissues of mice with EAE [112,113]. IL-18 exerts its pro-inflammatory effects by binding to the IL-18 receptor (IL-18R), activating NF-κB signaling, and promoting Th1 differentiation, which leads to IFN-γ induction. Nevertheless, the precise mechanisms by which IL-18 regulates MS and EAE progression remain incompletely understood [111,114,115].

*IL-1β*

**Figure 8:**
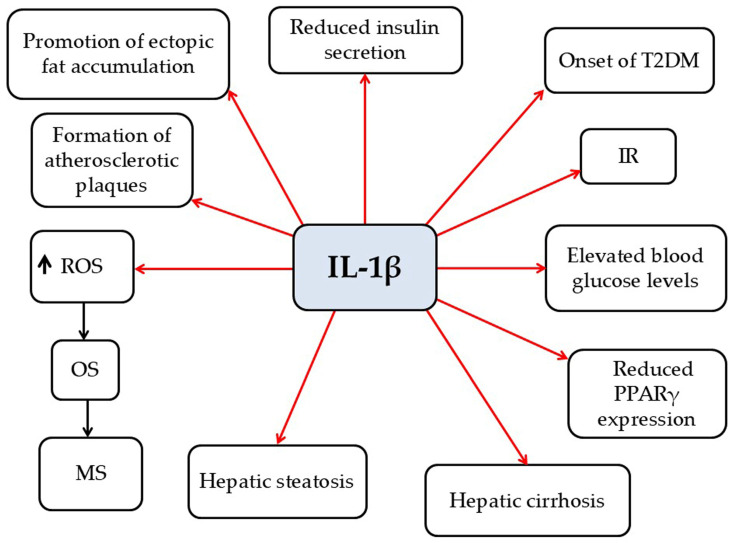
Harmful actions of IL-1β in chronic inflammation and OS associated with obesity and MS.

IL-1β is a pro-inflammatory cytokine primarily secreted by M1 macrophages [7]. In the context of obesity, IL-1β contributes to multiple pathologic processes, including (a) promoting ectopic fat accumulation, (b) elevating blood glucose levels, (c) impairing insulin secretion, (d) inducing IR, (e) increasing the risk of T2DM, (f) facilitating atherosclerotic plaque formation, (g) causing hepatic steatosis, (h) promoting liver cirrhosis, (i) suppressing PPARγ expression, (j) increasing cytokine and chemokine expression, and (k) inducing OS [7] (Figure 8). IL-1β mediates neuroinflammation by enhancing innate immune responses during MS pathophysiology [116]. A hallmark of EAE or MS progression is the disruption of the BBB and BSCB, which facilitates IL-1β release, tissue permeation, and subsequent neuroinflammation [117,118,119]. Notably, mice deficient in IL-1β or the IL-1R exhibit resistance to EAE [120,121]. Although the precise mechanisms by which IL-1β contributes to EAE or MS remain unclear, evidence indicates that it plays a key role in driving neuroinflammation processes [119]. Elevated IL-1β levels appear to play a pivotal role in MS pathophysiology by activating microglia and astrocytes, disrupting the BBB, recruiting peripheral immune cells, and exacerbating demyelination and neuronal damage through the promotion of reactive T-cell responses. Consequently, targeting IL-1β signaling represents a promising therapeutic strategy to modulate neuroinflammation and potentially slow MS progression. Overall, IL-1β serves not only as a key mediator of neuroinflammation in MS but also as a potential biomarker of disease activity. 

#### 3.1.4. Anti-Inflammatory Cytokines in MS

*IL-10*

IL-10 is an anti-inflammatory cytokine that plays a crucial role in preventing inflammatory and autoimmune pathologies [122]. It is primarily produced by activated myeloid cells and lymphocytes, with lower levels produced by other cell types during inflammation [123]. IL-10 functions by dimerizing and binding to the extracellular domains of two IL-10R1 subunits within the IL-10 receptor complex, which is a tetramer composed of two IL-10R1 and two IL-10R2 subunits [123]. The IL-10R1 subunit associates with JAK1, whereas IL-10R2 is linked to TYK2 [123]. Activation of the IL-10 receptor complex triggers phosphorylation and activation of STAT1, STAT3, STAT5, and SOCS1/3, ultimately inhibiting NF-κB-mediated signaling [123]. IL-10 suppresses the production of pro-inflammatory cytokines, including IL-1β, IL-6, IL-12, IL-18, and TNF-α, while promoting the synthesis of anti-inflammatory mediators such as the IL-1β receptor antagonist [123]. Its anti-inflammatory effects have been demonstrated in experimental models of MS, where it attenuates neuroinflammation [123,124]. In EAE mouse models, IL-10-deficient mice developed more severe disease compared to wild-type mice, whereas mice overexpressing IL-10 were resistant to EAE [125,126]. These findings indicate that IL-10 functions as a protective and immunoregulatory cytokine in MS, maintaining immune balance by suppressing pro-inflammatory cytokine production, inhibiting T-cell responses, and promoting regulatory T-cell activity. Reduced IL-10 levels or signaling are associated with increased disease severity, highlighting its critical role in preventing inflammation and tissue damage. Consequently, strategies that enhance or mimic IL-10 activity may offer a promising therapeutic approach for managing MS progression. 

### 3.2. Activation of the NLRP3 Inflammasome in Obesity and MS

Inflammasomes are large cytoplasmic multiprotein complexes that function through PRRs to promote the maturation and secretion of pro-inflammatory cytokines, including IL-1β and IL-18, which are key mediators of inflammation [25,127]. Structurally, inflammasomes are composed of three main components: (a) a sensor protein, such as members of the NLR family; (b) an adaptor protein ASC (also known as PYCARD), which contains a caspase recruitment domain; and (c) caspase-1 [128]. TLRs and NLRs are members of the PRR family, recognizing PAMPs and DAMPs, respectively [128]. Activation of PRR initiates inflammasome assembly and triggers NF-κB signaling. The adaptor protein ASC links NLR to caspase-1, enabling complex formation [128]. Activation of the NLRP3 inflammasome occurs when PAMPs or DAMPs engage NLR receptors in response to various metabolic disturbances, including lysosomal disruption, release of mtDNA, increased ROS, and elevated intracellular calcium (Ca^2+^) levels [128]. This leads to NLR oligomerization through PYD or CARD interactions, followed by caspase-1 activation, which promotes the maturation and secretion of IL-1β and IL-18 [128]. Caspase-1 activation also induces pyroptosis, a form of programmed inflammatory cell death [128]. 

NLRP3 inflammasomes belong to the NLR family and are composed of an LRR domain, a nucleotide-binding site (NBS), and a PDC-3 domain [128]. Esser et al. found elevated expression of NLRP3 and IL-1β in macrophages infiltrating the visceral fat of obese individuals with MetS, compared with obese individuals without MetS [129]. In obesity, NLRP3 inflammasomes are activated by an excess of metabolic DAMPs such as ATP, glucose, FAs, ceramides, ox-LDL, crystallized uric acid, cholesterol crystals, and monosodium urate. Additionally, pro-inflammatory adipokines such as leptin, resistin, and TNF-α further promote NLRP3 activation, resulting in caspase-1-mediated maturation and secretion of IL-1β and IL-18 [16,130]. Palmitic acid, a saturated fatty acid, activates the NLRP3-PYCARD inflammasome through mechanisms involving mitochondrial ROS, AMPK inhibition, and the ULK1-dependent autophagy signaling pathway, ultimately leading to caspase-1 and the production of IL-1β and IL-18. These findings support a link between high-fat diets and inflammation [131]. In contrast, oleic acid, an unsaturated fatty acid, counteracts the inflammatory effects of palmitic acid by enhancing AMPK activation and reducing ER stress [132]. Long-chain PUFAs, such as omega-3 fatty acids, also inhibit caspase-1 activation via their receptor GPR120, which recruits β-arrestin-2 to form a complex that interacts with and inhibits NLRP3 inflammasome activation [133]. Conversely, cholesterol crystals activate NLRP3 through lysosomal destabilization [134], while ox-LDL promotes inflammasome activation via NF-κB signaling [135]. Hyperglycemia can also activate the NLRP3 inflammasome in human adipose tissue through upregulation of TXNIP, leading to increased IL-1β expression and contributing to IR [136]. Elevated glutamate levels during glucose deprivation and hypoxia induce ER stress, raise intracellular calcium (Ca^2+^) levels, and further enhance TXNIP expression [137]. LPSs, potent endotoxin PAMPs, are increased in conditions such as obesity and T2DM due to alterations in gut microbiota and enhanced intestinal permeability. LPS are taken up by macrophages and adipocytes, where they activate NLRP3 inflammasomes and pro-IL-1β production through TLR4- and NF-κB-dependent pathways, initiating robust inflammatory responses [138]. Additionally, ATP, beyond its role as an intracellular energy carrier, is a major activator of the NLRP3 inflammasome via the NF-κB signaling [139]. NLRP3 inflammasomes also play a significant role in MS pathophysiology [25]. Increased expression of NLRP3 and IL-1β has been found in MS lesions, accompanied by elevated serum levels of ASC, caspase-1, and IL-18 [140,141]. In EAE models, both NLRP3 mRNA and protein levels are upregulated [111,142], whereas Nlrp3−/− mice display reduced Th1 and Th17 lymphocyte infiltration in the spinal cord and peripheral lymphoid tissues, along with markedly attenuated disease severity compared with wild-type mice [111,143]. Moreover, Yu et al. reported that TSLP-deficient mice (Tslpr−/− mice) exhibit decreased NLRP3 expression and lower EAE scores [144]. Thus, the activation of the NLRP3 inflammasome represents a key mechanistic link between obesity and neuroinflammation in MS, mediating inflammatory responses to metabolic disturbances. In obesity, NLRP3 activation within adipose tissue increases the production of pro-inflammatory cytokines, which can enter the circulation and contribute to CNS neuroinflammation, thereby exacerbating MS symptoms. Targeting the NLRP3 inflammasome pathway offers a promising strategy to reduce both systemic and CNS inflammation. Inhibiting NLRP3 activation has the potential to alleviate obesity-related complications, MS, and a range of other inflammatory diseases. Consequently, modulation of this pathway may provide meaningful benefits in controlling disease severity and progression in MS.

### 3.3. Gut Microbiota Dysbiosis in Obesity and Multiple Sclerosis (MS)

Significant differences have been identified between the gut microbiota of obese individuals and those of normal-weight individuals [16], reflecting alterations in both the composition and functional capacity of the host microbiome [145]. The gut microbiota in obesity can increase intestinal and BBB permeability [16,146,147]. As a result, LPSs derived from the outer membrane of Gram-negative bacteria can translocate across the intestinal mucosa and subsequently cross the BBB, where they act on astrocytes and microglia [146,147,148,149,150]. This microbial translocation contributes to shifts in the Treg/Th17 cell balance within the CNS, thereby promoting neuroinflammation [151]. In obesity, certain bacterial groups, such as Archaea, are present in greater abundance, whereas others, including members of the Firmicutes and Bacteroidetes phyla, are reduced or absent [152]. In MS, the presence of specific gut microbial taxa, such as Fusobacteria, has been associated with an increased risk of disease relapse [153]. However, therapeutic strategies for modifying the gut microbiota in MS, including probiotics and fecal microbiota transplantation, have thus far shown limited success [154]. Furthermore, MS patients have been found to exhibit reduced levels of propionic acid in both feces and blood samples [155]. Exogenous administration of propionic acid as an adjunct therapy in MS has been found to significantly reduce Th1 cell activity and enhance Treg function, resulting in decreased disability and fewer disease relapses [155]. These findings suggest that many of the detrimental effects associated with obesity and MS progression may be mediated through alterations in the gut microbiome. Conversely, several therapeutic approaches for these conditions may, at least in part, exert their benefits by modulating this microbiome–immune axis, ultimately influencing neuronal function and systemic inflammatory profiles.

## 4. OS in Obesity and MS

OS in obesity arises primarily through two main mechanisms. The first involves chronic inflammation in adipose tissue, where excessive production of pro-inflammatory cytokines such as TNF-α, IL-6, and IL-1β stimulates mitochondrial and peroxisomal oxidative phosphorylation, leading to the generation of FRs, mitochondrial DNA damage, ATP depletion, and lipotoxicity [7,60]. This chronic inflammation is further amplified by activation of PGE2, COX-2, and MAPK pathways [156]. Moreover, obesity is associated with reduced expression of PPAR-γ due to the influence of FAs and their metabolites, impairing its ability to upregulate antioxidant genes and suppress pro-inflammatory mediators [157]. The second pathway of OS in obesity involves NADPH oxidase formation, which is promoted both by pentose phosphate pathway signaling and by increased NADPH oxidase expression in macrophages [156]. These enzymes transfer electrons from NADPH to molecular oxygen, generating superoxide anion radicals (O_2_^•−^), which are subsequently converted to H_2_O_2_ [156]. H_2_O_2_ is further detoxified into water and oxygen by antioxidant enzymes such as catalase (CAT) and glutathione peroxidase (GPx); in cases of antioxidant enzyme deficiency, ROS accumulate [156,158]. Consequently, ROS overproduction in obesity arises from both NADPH oxidase activity and mitochondrial oxidative phosphorylation. Within mitochondria, molecular oxygen (O_2_) is reduced to H_2_O through the flavin mononucleotide and ubiquinone cycle, producing both O_2_^•−^ and H_2_O_2_ [159]. Excessive ROS generation leads to OS, which is reflected by increased levels of MDA, a biomarker of cellular damage and lipid peroxidation [156].

**Figure 9:**
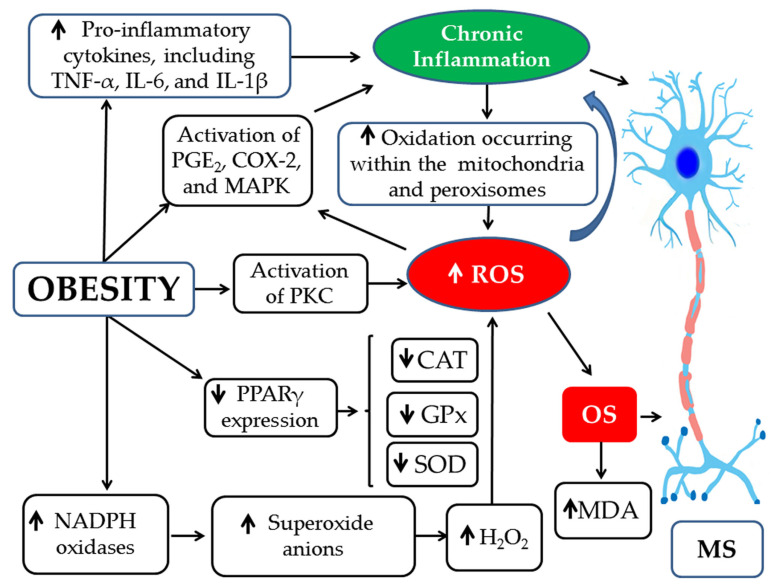
Schematic representation of the interaction between obesity, chronic inflammation, and oxidative stress in the pathogenesis of multiple sclerosis (MS), emphasizing that OS in obesity arises via two mechanisms. The first mechanism originates from chronic inflammation in adipose tissue, where excessive secretion of pro-inflammatory cytokines such as TNF-α, IL-6, and IL-1β stimulates mitochondrial and peroxisomal oxidative phosphorylation, resulting in ROS production. This inflammatory state is further amplified by activation of PGE2, COX-2, and MAPK pathways. Additionally, obesity is associated with reduced expression of PPAR-γ due to the influence of FAs and their metabolites, impairing the induction of antioxidant enzymes, including catalase, glutathione peroxidase, and superoxide dismutase. The second mechanism of OS in obesity involves NADPH oxidase formation. These enzymes transfer electrons from NADPH to molecular oxygen, generating superoxide anion radicals (O_2_^•−^), which are subsequently converted to H_2_O_2_. H_2_O_2_ is then detoxified into H_2_O by antioxidant enzymes such as CAT and GPx. In the absence of sufficient antioxidant defenses, ROS accumulate. Consequently, ROS overproduction in obesity arises from both NADPH oxidase activity and mitochondrial oxidative phosphorylation. This OS is characterized by elevated MDA levels and increased lipid peroxidation, which promote chronic low-grade inflammation. Moreover, OS further activates PGE2, COX-2, and MAPK pathways, amplifying inflammatory responses. Together, chronic inflammation and OS contribute to the pathogenesis of MS.

In MS, neuroinflammation activates the MAPK signaling pathway within the nuclei of macrophages and dendritic cells, leading to the overproduction of pro-inflammatory cytokines, including TNF-α, IFN-γ, IL-1β, IL-12, IL-6, and IL-23 [160]. Concurrently, activation of the transcription factor NF-κB in CNS cells such as T lymphocytes, macrophages, microglia, astrocytes, and oligodendrocytes amplifies neuroinflammation and drives MS pathogenesis [161]. Neuroinflammation signaling also activates JNK pathways, resulting in demyelination and neuronal apoptosis [162], while disruption of the PI3K/AKT pathway further contributes to disease exacerbation [163]. Neuroinflammation promotes the excessive production of ROS and NO by glial cells and activates the arachidonic acid pathway in the CNS via COX and LOX, leading to OS [164,165]. OS can damage DNA, lipids, and proteins, ultimately causing cell death [166]. It can also impair mitochondrial function and sodium-potassium pump activity, reducing ATP production, causing intracellular potassium accumulation, and triggering apoptosis [167]. Furthermore, overproduction of ROS and NOS in neural cells contributes to myelin sheath damage and compromises BBB integrity [168,169,170] (Figure 9).

## 5. Antioxidant Compounds in the Management of Multiple Sclerosis

A key regulator of the antioxidant response is Nrf2 [171]. Under basal conditions, Nrf2 is sequestered in the cytoplasm by Keap1, which targets it for degradation via the ubiquitin-proteasome system [171]. Under OS, Nrf2 dissociates from Keap1 and translocates to the nucleus, where it binds to AREs to induce the transcription of genes encoding antioxidant enzymes and detoxification proteins. This process protects cells from ROS-induced damage and helps maintain redox homeostasis [172]. The Nrf2 signaling pathway represents a promising target for enhancing antioxidant defenses in MS, as it regulates the expression of several antioxidant enzymes [173]. Moreover, the JNK and ERK pathways can phosphorylate Nrf2, promoting its translocation to the nucleus [174,175]. Endogenous antioxidant defenses, including enzymatic antioxidants such as glutathione peroxidase, catalase, SOD, and PON2, and free radical scavengers such as alpha-tocopherol (vitamin E) and glutathione [164,176]. Nrf2 regulates several key enzymes, including SOD, HO-1, GPxs, and catalase [172]. Catalase, a primary defense against ROS and OS, has been found at increased levels in the gray matter of MS patients compared to controls, suggesting a compensatory response to elevated OS [177]. Similarly, SOD serves as a primary defense against OS, and reduced SOD activity has been associated with excessive ROS production [172]. Obradovic et al. reported elevated SOD activity in the serum of MS patients, reflecting ongoing oxidative and inflammatory damage [178]. Additionally, the coenzyme CoQ10 may provide dose-dependent benefits in mitigating OS and inflammation in MS [179]. Several non-enzymatic compounds, including PUFA, glutathione, NAC, alpha-lipoic acid, melatonin, L-carnitine, and various polyphenols, including epigallocatechin, quercetin, curcumin, and resveratrol, have demonstrated protective effects against obesity-related metabolic disturbances [180] and may serve as adjunctive therapies in MS [181]. Similarly, trace elements such as Fe, Zn, Mn, and Cu may confer beneficial effects on obesity-related metabolic disorders, as imbalances of these elements have been found in patients with metabolic abnormalities [182]. They may also play a supportive role in MS management. However, their potential pro-oxidant effects and capacity to induce cellular damage should not be overlooked, particularly in the case of iron and copper, which can promote oxidation of cellular components and contribute to the formation of advanced glycation end products via Fenton reaction propagation [183,184]. Targeting inflammatory mediators represents a promising therapeutic strategy for MS, though the safety and efficacy of such interventions require further validation in clinical trials. Anti-inflammatory approaches include (a) IL-1R antagonists [7,185], (b) recombinant anti-IL-1β antibodies [7], (c) NF-κB inhibitors [7], (d) anti-TNF-α agents [7], (e) inflammasome-targeting compounds [127,186,187,188], and (f) thiazolidinediones, which act as potent and selective PPAR activators [7,186,187,188]. Additionally, anti-obesity drugs with anti-inflammatory and antioxidant properties may offer potential as adjunct therapies in MS management [189].

### 5.1. Antioxidants as Complementary Therapy in MS

#### 5.1.1. Vitamin D

**Figure 10:**
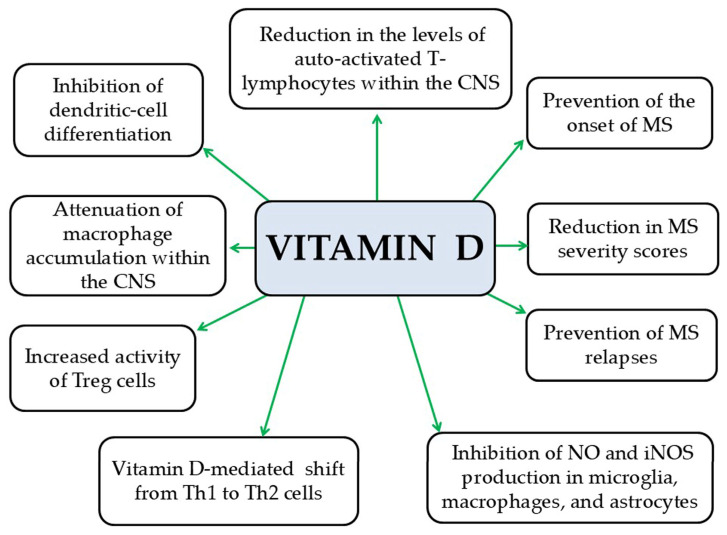
Schematic representation of the beneficial effects of vitamin D against MS.

Vitamin D reduces the number of activated autoreactive T lymphocytes in the CNS [190], which are responsible for attacking the myelin sheath and contributing to MS pathogenesis [191]. It also appears to inhibit the differentiation of dendritic cells in vitro [192,193], cells that play a key role in innate immune responses involved in MS [194]. Furthermore, vitamin D decreases macrophage accumulation in the CNS in EAE models, suggesting potential neuroprotective effects [195,196,197,198]. In parallel, vitamin D has been shown to enhance Treg activity in EAE models [199], which helps suppress autoreactive T lymphocytes [200]. Additionally, vitamin D promotes a shift from Th1 to Th2 cells [201], thereby altering T-cell cytokine profiles from pro-inflammatory Th1 cytokines such as TNF-α, IFN-γ, and IL-2 [201] to anti-inflammatory Th2 cytokines, including IL-4, IL-5, and IL-10 [202]. Moreover, vitamin D reduces NO production [203] and suppresses the iNOS pathway [204] in microglia, macrophages, and astrocytes in vitro [205]. This is particularly important, as NO contributes to BBB disruption, oligodendrocytes and axonal damage, and demyelination [206]. Overall, vitamin D exerts a protective role in MS onset, mitigates disease severity, and decreases relapse frequency [207,208] (Figure 10). However, the optimal dose of vitamin D supplementation as an adjunct therapy in MS remains uncertain. A significant knowledge gap also exists regarding the extent to which obesity alters the immunological and clinical effects of vitamin D in MS. Although obese patients may require higher vitamin D doses due to altered vitamin D metabolism and sequestration in adipose tissue, this has not been confirmed in clinical trials. Therefore, careful evaluation of the long-term safety of high-dose vitamin D supplementation in obese individuals with MS is essential, particularly since their pharmacokinetic profile may differ substantially from that of non-obese patients.

#### 5.1.2. Vitamin A

Vitamin A, particularly through its active metabolite retinoic acid, enhances the integrity of the BBB [209], thereby limiting the entry of peripheral pro-inflammatory mediators and immune cells into the brain parenchyma [181,210]. In addition, vitamin A exhibits anti-inflammatory and antioxidant effects within the CNS [181,211]. It suppresses the secretion of pro-inflammatory cytokines such as IL-1, IL-12, TNF-α, and NO [181,212].

**Figure 11:**
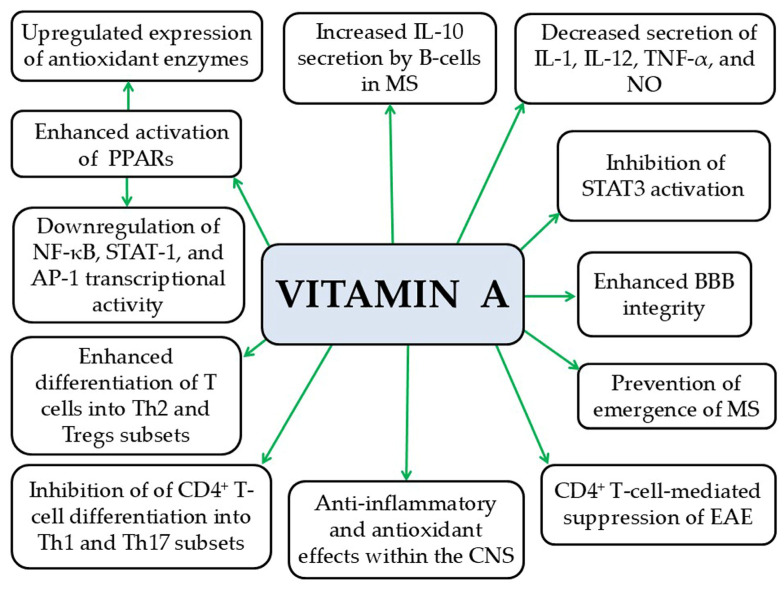
Schematic representation of the beneficial effects of vitamin A against MS.

Vitamin A also increases the in vitro secretion of the anti-inflammatory cytokine IL-10 by B cells in MS and enhances the production of antioxidant enzymes, thereby protecting the brain from OS [181,213]. Furthermore, in experimental models, vitamin A activates PPARs, modulating the phenotype of CNS macrophages and consequently reducing neuroinflammation, neuronal OS, and axonal demyelination [181]. In addition, preclinical studies show that vitamin A inhibits the transcription factors NF-κB and AP-1, both involved in T-cell activation, as well as STAT-1, a key regulator of neuroinflammation [181,214]. Vitamin A also promotes the differentiation of T cells into Th2 cells, which produce anti-inflammatory cytokines, an effect supported by preclinical studies [181,215]. Likewise, retinoic acid, the active metabolite of vitamin A, increases the number of Treg cells, which suppress autoreactive T lymphocytes that attack the myelin sheath and contribute to MS pathogenesis [181,216]. At the same time, vitamin A inhibits the differentiation of CD_4_^+^ T cells into Th1 and Th17 subsets, thereby protecting against neuroinflammation [181,216] (Figure 11). Consistent with these actions, vitamin A reduces the ability of CD_4_^+^ T cells to induce EAE and exerts a protective role in MS onset [181,216]. Despite preclinical evidence suggesting that vitamin A and its active metabolite, retinoic acid, may benefit MS by modulating T-cell responses and reducing neuroinflammation, the influence of obesity on these therapeutic outcomes remains poorly understood. To date, no studies have investigated how obesity may alter the immunomodulatory or neuroprotective effects of vitamin A in MS, which is crucial to establishing safe and effective supplementation strategies.

#### 5.1.3. Curcumin

**Figure 12:**
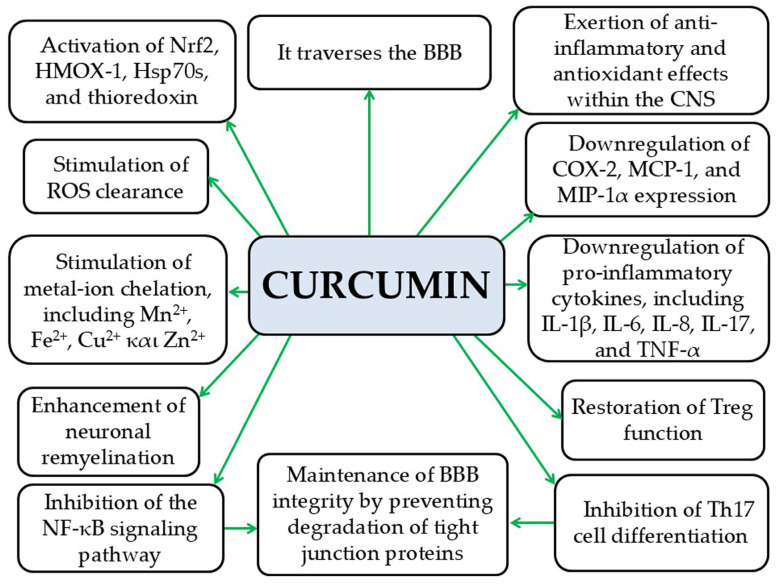
Schematic representation of the beneficial effects of curcumin against MS.

Curcumin is capable of crossing the BBB, where it protects the brain from inflammatory damage [217,218]. It prevents the degradation of tight junction proteins [219], thereby maintaining BBB integrity and limiting the infiltration of peripheral immune cells and inflammatory mediators into the brain parenchyma [181]. In addition, curcumin has been shown to promote neuronal remyelination in animal models of MS [220]. It also exhibits anti-inflammatory and antioxidant effects in the CNS, enhances the clearance of ROS, and chelates metal ions such as manganese, iron, copper, and zinc, as demonstrated in preclinical studies [181,221,222]. Additionally, curcumin inhibits the in vitro expression of pro-inflammatory cytokines such as IL-1β, IL-6, IL-8, IL-17, and TNF-α in the CNS, as well as COX-2, MCP-1, and MIP-1α [223,224]. It also suppresses NF-κB activation, thereby attenuating pro-inflammatory signaling pathways involved in the pathogenesis of both MS and EAE, as demonstrated in preclinical studies [181]. Likewise, curcumin inhibits the differentiation of Th17 cells, which play a key role in MS pathogenesis, according to preclinical research [225]. Furthermore, curcumin has been shown to restore Treg function [226], supporting the suppression of autoreactive T lymphocytes that attack the myelin sheath and drive MS development [227]. Curcumin also activates Nrf2, providing protection against OS, mitochondrial dysfunction, neuroinflammation, and neurodegeneration in MS, as demonstrated in preclinical studies [221,228]. Additionally, preclinical evidence indicates that curcumin exerts neuroprotective effects in neurodegenerative diseases by upregulating antioxidant systems, such as Hsp70s, HO-1, and thioredoxin, which are essential for maintaining mitochondrial ROS homeostasis [218,228,229] (Figure 12). However, despite these promising findings, curcumin’s poor bioavailability, limited solubility, and rapid metabolism and excretion significantly hinder its therapeutic potential, underscoring the need for improved pharmaceutical formulations and adjuvants to enhance its pharmacokinetics [230]. Moreover, its potent biological activity in vivo, combined with suboptimal pharmacokinetics, suggests that part of its effectiveness may involve actions within the gastrointestinal tract and modulation of the microbiota–gut–brain axis [230].

#### 5.1.4. Resveratrol

**Figure 13:**
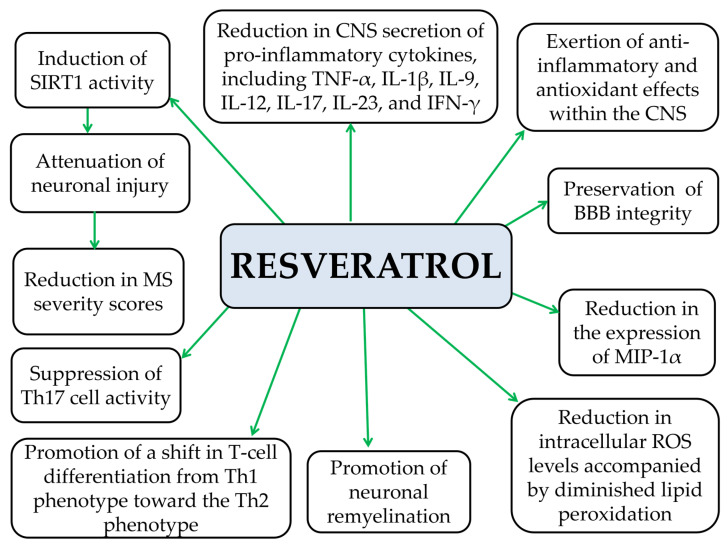
Schematic representation of the beneficial effects of resveratrol against MS.

Preclinical evidence indicates that resveratrol enhances BBB integrity in EAE mouse models, thereby limiting the infiltration of peripheral pro-inflammatory mediators and immune cells into the brain parenchyma [231]. It also exerts potent anti-inflammatory and antioxidant effects within the CNS, reducing ROS and pro-inflammatory cytokines such as TNF-α, IL-1β, IL-9, IL-12, IL-17, IL-23, and IFN-γ [181,228]. Moreover, some preclinical studies show that resveratrol suppresses the expression of MIP-1α [228] and inhibits Th17 cell responses, which are central to MS pathogenesis [232]. At the same time, resveratrol promotes a shift from Th1 cells toward Th2 cells, thereby altering T lymphocyte cytokine production from pro-inflammatory mediators such as TNF-α, IFN-γ, and IL-2 to anti-inflammatory cytokines, including IL-4, IL-5, and IL-10 in EAE models [232]. Moreover, resveratrol activates SIRT1, an NAD^+^-dependent deacetylase whose overexpression appears to exert neuroprotective effects in the CNS [181,232,233]. It also enhances neuronal remyelination in EAE models [228]. Through its combined anti-inflammatory, antioxidant, and anti-apoptotic actions, resveratrol reduces neuronal damage and attenuates the severity of MS [228] (Figure 13). Despite extensive resveratrol research in obesity and MetS models, there is a notable lack of preclinical studies investigating its effects in combined obesity–MS models. This represents a critical gap in understanding how resveratrol might influence MS pathogenesis in the context of metabolic dysfunction. Without such combined-model data, predicting patient safety, treatment efficacy, and optimal dosing of resveratrol in obese MS patients remains challenging, posing significant implications for the design of future clinical trials.

#### 5.1.5. Quercetin

**Figure 14:**
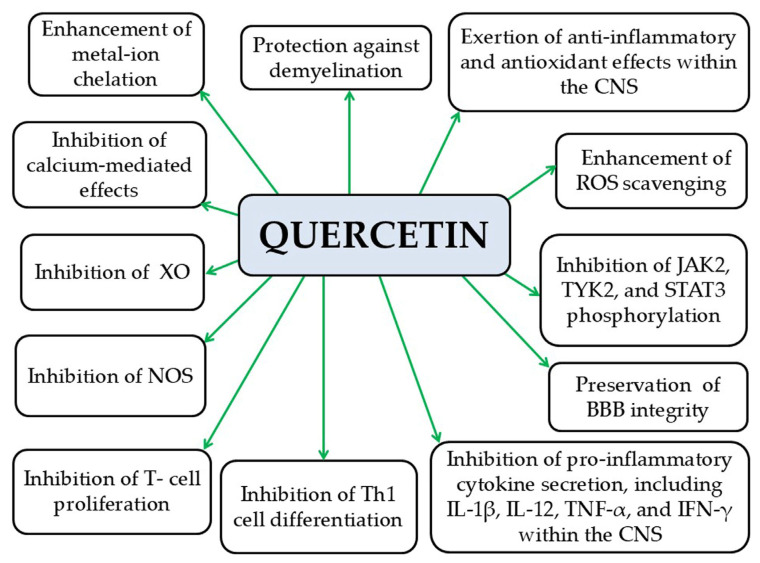
Schematic representation of the beneficial effects of quercetin against MS.

Quercetin strengthens the BBB, limiting the infiltration of peripheral pro-inflammatory substances and immune cells into the brain parenchyma [234]. Preclinical studies indicate that quercetin exerts anti-inflammatory and antioxidant properties in the CNS by promoting ROS clearance, inhibiting the secretion of pro-inflammatory cytokines such as IL-1β, IL-12, and TNF-α, and chelating metal ions [181,234]. Additionally, quercetin has been shown to suppress NOS activity in macrophages and astrocytes and inhibits the proliferation of autoreactive T cells, which attack the myelin sheath and contribute to MS pathogenesis [234]. Quercetin also inhibits the differentiation of Th1 helper T cells, thereby reducing demyelination and promoting remyelination [234]. However, its poor pharmacokinetics may limit its clinical applicability, a challenge shared with other naturally derived compounds such as resveratrol, which, despite high membrane permeability, exhibits low bioavailability due to rapid phase II metabolism (even at the intestinal level) and structural instability under UV light, high temperature, pH fluctuations, and oxidative enzymes [235,236]. Additionally, quercetin reduces IFN-γ production and inhibits calcium-mediated signaling in CNS cells, exerting neuroprotective effects [181]. It also inhibits xanthine oxidase, an enzyme implicated in axonal and myelin damage in EAE models [181]. Quercetin also inhibits the phosphorylation of JAK2, TYK2, and STAT3, thereby exerting anti-inflammatory and anti-apoptotic effects in the CNS [181] (Figure 14). While preclinical studies support the anti-inflammatory and antioxidant effects of quercetin in MS models, there remains a pronounced gap in research exploring its role in the context of MS coexisting with obesity or MetS. Given that obesity can exacerbate neuroinflammation and alter immune responses, understanding the interplay between quercetin, MS pathogenesis, and obesity-associated metabolic disturbances is essential. Addressing this knowledge gap is crucial for predicting therapeutic efficacy, optimizing dosing strategies, ensuring patient safety, and informing the design of future preclinical and clinical studies.

## 6. Conclusions

MS is a degenerative disease characterized by a complex and multifactorial pathophysiology, posing significant challenges for affected individuals. Consequently, effective treatment and prevention of disease progression remain major clinical and research priorities. Growing evidence indicates that MS progression shares common pathophysiological pathways with obesity and related metabolic disorders [30]. These overlapping pathways contribute to key disease manifestations, including chronic inflammation, OS, IR, cellular degeneration, and apoptosis [30]. In the brains of obese individuals, elevated levels of ROS are generated as a result of persistent neuroinflammation. These high ROS levels impair brain mitochondrial ATP production, which is essential for the proper function of neurons and glial cells. Moreover, excessive ROS cause damage to the phospholipid membranes of neural cells. Both chronic neuroinflammation and OS contribute to the pathophysiology of MS, and the two processes interact and exacerbate one another. OS arises from an imbalance between ROS and the antioxidant defense system, wherein ROS levels exceed antioxidant capacity, leading to oxidative damage. OS is also associated with inflammasome activation, gut microbiota dysbiosis, cytokine-induced synaptic hyperexcitability, abnormal iron accumulation in the brain, and microglial activation, all of which contribute to neuronal injury. OS biomarkers measured in serum or CSF may hold diagnostic and prognostic value in MS. MS manifests in distinct forms, including RRMS, PPMS, and SPMS. The impact of obesity on these subtypes and on overall disease progression remains an important area of investigation, as the obesity-associated metabolic and inflammatory factors may differentially influence relapse rates, neurodegeneration, and long-term outcomes. In RRMS, obesity is associated with elevated serum and CSF levels of pro-inflammatory cytokines such as IL-6 and adipokines, including leptin, resistin, and chemerin, alongside reduced levels of the anti-inflammatory adipokine adiponectin [237]. This heightened inflammation state is thought to result from metabolic and immunological alterations associated with obesity. Moreover, higher BMI correlates with an increased risk of relapses and greater disability in MS patients, as reflected by EDSS scores of 3 and 4 [238]. In individuals with CIS, obesity predicts faster conversion to definite MS and higher annual relapse rates [239]. In patients with PPMS or SPMS, obesity at the time of diagnosis is associated with faster accumulation of disability over time, as reflected by EDSS scores [240]. Moreover, sustained elevated BMI from early adulthood through the time of diagnosis correlates with a higher risk of long-term disability progression, suggesting that prolonged obesity may exacerbate disease severity [241]. Although growing evidence highlights important links between obesity and potentially worse MS progression, substantial gaps remain in understanding the underlying mechanisms and the consistency of these effects, particularly in males and across different disease stages. Much of the existing research focuses on RRMS or CIS, leaving PPMS and SPMS comparatively understudied, particularly regarding long-term disease progression and treatment considerations. Future targeted research is therefore essential to elucidate how obesity may differentially influence disease onset, progression, and outcomes across MS subtypes [242,243,244]. Table 1 summarizes the effects of anti-inflammatory and pro-inflammatory adipokines and cytokines on inflammation, OS, obesity, and MS. Although demyelination and inflammatory processes are partially interlinked (given that MS involves both central and peripheral inflammation), they are not directly or consistently correlated. Consequently, correlations between adipokines and inflammatory markers cannot be established with complete certainty at this time. This limitation highlights the need for further research to clarify these complex interactions. Anti-inflammatory strategies show considerable promise for slowing MS progression. These approaches include blocking the IL-1 receptor, inhibiting IL-1β or NF-κB signaling, modulating TNF/TNFR1 activity [245], regulating inflammasome activation, and stimulating PPAR pathways. By targeting these inflammatory mediators, such interventions aim to suppress neuroinflammation and correct immune dysregulation, potentially improving clinical outcomes and mitigating long-term disease progression. Despite their promise, these therapeutic strategies require further investigation, both fundamental studies to elucidate mechanisms and biomarkers and translational studies for drug development and clinical trials, because the immune system in the brain is complex and tightly regulated. 

Substantial reduction in obesity through individualized dietary strategies, modulation of the gut microbiota, and targeted nutritional supplementation may offer important benefits for patients with MS [246]. Such approaches typically involve reducing saturated fat intake together with a higher consumption of plant-based foods, especially fruits and vegetables rich in antioxidants, including polyphenols, vitamins, and trace elements [246]. Complementary supplementation with compounds of high antioxidant bioavailability may further enhance neuroprotection and anti-inflammatory effects [246]. Together, these nutritional interventions could complement anti-inflammatory symptomatic treatments, potentially contributing to improved MS management and reduced relapse frequency. Moreover, anti-obesity drugs with demonstrated anti-inflammatory and antioxidant properties, such as orlistat, liraglutide, semaglutide, and tirzepatide [247], may modulate key mechanisms underlying MS. Emerging evidence suggests that several anti-diabetic and weight-loss medications, particularly the GLP-1 receptor agonists semaglutide, dulaglutide, and liraglutide; the SGLT2 inhibitor empagliflozin; and the biguanide metformin, may be inversely associated with MS risk of progression [248]. However, these observations require confirmation through rigorous, well-designed prospective studies. Further research is also needed to evaluate the safety and efficacy of combining anti-obesity medications with anti-inflammatory and antioxidant properties alongside novel antioxidant compounds as complementary therapies in MS. Polyphenols represent a particularly promising adjunctive strategy at the interface of obesity and MS due to their potent antioxidant and anti-inflammatory activities, which can counteract the chronic low-grade inflammation characteristic of obesity. They may also reduce the risk and severity of obesity-related comorbidities, including mechanisms that influence MS pathogenesis and progression [30,248]. By inhibiting key pro-inflammatory pathways, such as NF-κB and MAPK signaling, while simultaneously promoting anti-inflammatory responses, polyphenols provide a biologically plausible mechanism for modulating neuroinflammatory processes [30]. Although current findings are encouraging, further mechanistic studies and well-designed clinical trials are essential to clarify their therapeutic potential, optimal dosing, and long-term safety. Taken together, the available evidence positions polyphenols as a compelling target for future research and a potential complementary approach in managing both obesity and MS.

## Figures and Tables

**Table 1:** Comparative effects of various anti- and pro-inflammatory adipokines and cytokines on inflammation, OS, obesity, and MS.

Adipokines and Cytokines	Inflammation	OS	Obesity	MS
**Anti-inflammatory adipokines**				
Adiponectin	It suppresses NF-κB activity and reduces TNF-α and IL-6 secretion from macrophages [7].	It decreases ROS production and OS [7].	Clinical studies show low adiponectin baseline levels in obese patients [7].	Clinical studies demonstrate an “adiponectin paradox”, where low adiponectin baseline levels may be a sign of a less active MS disease [41], and high adiponectin overall levels may predict worse disease progression [38].
Apelin	It suppresses iNOS and IL-6 production and upregulates arginase-1 and IL-10 in N9 microglial cells [43].	It promotes the synthesis of antioxidant enzymes and inhibits FA oxidation and reduces OS [7].	It promotes the differentiation and metabolic activity of brown adipocytes, induces the browning of white adipose tissue, and inhibits lipogenesis and lipolysis [7]. Clinical studies show high apelin serum levels in obese patients [7].	Preclinical studies indicate that apelin can promote remyelination in MS research [46,47]. Clinical studies showed conflicting findings on apelin levels in MS patients. Tehrani et al. found lower serum apelin levels in women with early-stage RRMS than in healthy individuals. Apelin levels were positively linked to higher EDSS scores and more relapses [36]. In contrast, Alpua et al. found higher apelin levels in RRMS patients compared to controls, though they did not find a link to MS severity or duration [44].
**Pro-inflammatory adipokines**				
Leptin	It upregulates TNF-α and IL-6 from macrophages [7] and promotes the proliferation of Th1 and Th17 cells [50,51].	It promotes OS by increasing FA oxidation and inflammation [7].	Serum leptin levels are directly correlated with the mass of the adipose tissue [49]. In obesity, leptin resistance occurs, causing elevated serum leptin levels that fail to reduce appetite [53]. Leptin resistance is associated with impaired BBB function and decreased leptin transport across the BBB [53].	A preclinical study demonstrated that removing leptin receptors attenuated leukocyte infiltration into the CNS and improved the integrity of the BBB [56]. Clinical studies showed that serum and CSF leptin levels are increased in patients with RRMS, particularly during the acute phase of MS [54,55].
Visfatin	It upregulates the expression of chemokines such as CCL2, CXCL2, and CXCL8; upregulates the expression of adhesion molecules such as ICAM-1 and VCAM-1; and induces the release of pro-inflammatory cytokines such as IL-1β, IL-6, and TNF-α [7].	It promotes OS through the inflammatory NF-κB signaling pathway [7].	Elevated visfatin levels are often found in people with obesity, particularly those with central obesity and metabolic issues such as IR and T2DM [7].	It increases the synthesis of pro-inflammatory mediators such as IL-1β, IL-6, and iNOS in microglial cells in response to LPS stimulation [58]. There are increased visfatin levels in MS patients [59], particularly in those with RRMS, and these levels correlate positively with TNF-α and negatively with FoxP3 mRNA in T cells [60].
Resistin	It promotes the activation of pro-inflammatory cytokines, such as IL-1β, IL-6, and TNF-α, and upregulates several adhesion molecules [7].	It promotes OS and inhibits endothelial nitric oxide synthase (eNOS) expression [7].	In animal models, it promotes IR; however, there are inconsistent findings regarding the association between resistin levels and obesity, IR, and T2DM [7].	Serum resistin levels are significantly elevated in MS patients compared to healthy individuals [61]. This elevation is observed alongside increased levels of other inflammatory markers, such as IL-1β, TNF-α, and CRP [61]. In patients with RRMS, the higher serum resistin levels correlate with reduced Treg activity, which can potentially exacerbate the negative progression of the disease [41].
**Pro-inflammatory cytokines**				
TNF-α	It induces chronic inflammation via TNFR1 signaling and promotes immune regulation via TNFR2 signaling [81], stimulates MCP-1 and IL-6 secretion from preadipocytes [86], inhibits adiponectin synthesis [87], and activates the NF-κB pathway [87].	It promotes ROS production, including superoxide anion radical [7].	Elevated TNF-α levels are strongly associated with obesity and IR [7]; they increase FFA release from adipocytes [87], reduce insulin action on peripheral glucose uptake [7], and increase lipolysis in adipocytes [7].	There is a correlation between increased TNF-α levels and active MS, disease severity, and progression [10]. SNPs in the TNFR1 gene (TNFRSF1A) have been linked to increased MS risk [91]. In EAE mouse models, TNFR1 deficiency leads to protection or milder disease [94,95], while TNFR2 deficiency results in more severe disease [96,97,98].
IL-6	It induces the acute-phase response of inflammation [7,99,100].	It promotes ROS production by activating JAK1/STAT3 and MAPK pathways [99].	Elevated serum IL-6 levels are found in obese individuals [63,101]. High expression of IL-6 in the hypothalamus suggests a role for IL-6 in regulating appetite and food intake [63,101].	IL-6-deficient mice exhibit resistance to EAE [102,103].
IL-1β	It increases the expression of cytokines and chemokines [7].	It promotes mitochondrial OS and calcium release [7].	It promotes ectopic fat accumulation, increases blood glucose levels; causes IR, induces T2DM, contributes to the formation of atherosclerotic plaques and hepatic steatosis, and downregulates PPARγ expression [7]. Its blockade increases insulin secretion and reduced insulin requirements clinically [7].	IL-1β plays an important role in neuroinflammation and the development of EAE and MS [119]; mice deficient in IL-1β or IL-1R exhibit significant resistance to EAE [120,121].
**Anti-inflammatory cytokines**				
IL-10	It prevents inflammatory and autoimmune pathological conditions [122] and binds to its IL-10R complex, activating the STAT1, STAT3, STAT5, and SOCS1/3 signaling pathway. As a result, IL-10 signaling leads to inhibition of NF-κB-mediated transcription; suppresses the production of pro-inflammatory cytokines like IL-1β, IL-6, IL-12, IL-18, and TNF-α; and promotes the production of IL-1Ra [123].	It has antioxidant effects by the activation of PI3K signaling [237].	It is paradoxically upregulated in obesity and IR [238]. It seems to play a complex role in metabolic conditions and obesity despite its anti-inflammatory nature, as its ablation improves IR, protects against diet-induced obesity, and promotes the browning of white adipose tissue [239].	It reduces neuroinflammation in experimental models of MS [123,124]; IL-10-deficient mice develop more severe EAE compared to wild-type mice, whereas those overexpressing IL-10 exhibit resistance to EAE [125,126].

## Data Availability

No new data were created or analyzed in this study.

## Abbreviations

AIM: macrophage apoptosis inhibitor; AMPK: AMP-activated protein kinase; AP-1: activator protein 1; AREs: antioxidant response elements; BBB: blood–brain barrier; BMI: body mass index; BP: blood pressure; BSCB: blood–spinal cord barrier; CARD: caspase recruitment domain; CAT: catalase; CCL2: chemokine (C-C motif) ligand 2; CCL5-RANTES: C-C motif chemokine ligand 5-regulated upon activation, normal T-cell expressed and secreted; CCRL2: C-C chemokine receptor-like 2; CD4^+^ T lymphocytes: also known as helper T cells; CIS: clinically isolated syndrome; CMKLR1: chemokine-like receptor 1; CNS: central nervous system; CSF: cerebrospinal fluid; COX-2: cyclooxygenase-2; CRP: C-reactive protein; Cu: copper; CVDs: cardiovascular diseases; Cu^2+^: copper cation; CXCL2: chemokine (C-X-C motif) ligand 2 (also known as interferon-gamma-inducible protein 10); CXCL8: chemokine (C-X-C motif) ligand 8; DAMPs: damage-associated molecular patterns; EAE: experimental autoimmune encephalomyelitis; EDSS: Expanded Disability Status Scale; ER: endoplasmic reticulum; FA: fatty acid; FABP-4: fatty acid-binding protein 4; Fe^2+^: ferrous cation; FFAs: free fatty acids; FRs: free radicals; GLP-1: glucagon-like peptide-1; GPCRs: G protein-coupled receptors; GPR1: G protein-coupled receptor 1; GPR120: G-protein-coupled receptor 120; gp130: glycoprotein 130; GPx: glutathione peroxidase; HMGB1: high-mobility group box 1 protein; HMOX-1: heme oxygenase 1; HO-1: heme oxygenase-1; H_2_O_2_: hydrogen peroxide; Hsp70s: heat shock proteins 70; ICAM-1: intercellular adhesion molecule 1; IL-1β: interleukin-1β; IL-6: interleukin-6; IL-9: interleukin-9; IL-10: interleukin-10; IL-12: interleukin-12; IL-17: interleukin-17; IL-18: interleukin-18, also known as interferon-gamma (IFN-γ); IL-23: interleukin-23; IL-1Ra: Interleukin-1 receptor antagonist; IL-10R: interleukin-10 receptor; ILC2: type 2 innate lymphoid cells; IL-6: interleukin-6; INF-γ: interferon-gamma; iNKT: invariant natural killer T; iNOS: inducible nitric oxide synthase; IR: insulin resistance; JAK1/STAT3: janus kinase 1/signal transducer and activator of transcription 3; JKA2: Janus kinase 2; Keap1: Kelch-like ECH-associated protein 1; LOX: Lipoxygenase; LPS: lipopolysaccharide; LRR: leucine-rich repeat; MAPK: mitogen-activated protein kinase; MCP-1: monocyte chemoattractant protein-1; MDA: malondialdehyde; MetS: metabolic syndrome; MIP-1α: macrophage inflammatory protein-1α; MMP-9: matrix metalloproteinase-9; Mn^2+^: manganous cation; MS: multiple sclerosis; mtDNA: mitochondrial DNA; NAC: N-acetylcysteine; NADPH: nicotinamide adenine dinucleotide phosphate; NAFLD: non-alcoholic fatty liver disease; NBS: nucleotide-binding site; NF-κB: nuclear factor-kappa B; NLR: NOD-like receptor; NO: nitric oxide; Nrf2: nuclear factor erythroid 2-related factor 2; NOS: nitric oxide synthase; OS: oxidative stress; ox-LDL: oxidized low-density lipoproteins; PAI-1: plasminogen activator inhibitor-1; PAMPs: pathogen-associated molecular patterns; PBMCs: peripheral blood mononuclear cells; PDC-3: pyrin domain-containing component; pDCs: plasmacytoid dendritic cell; PGE_2_: prostaglandin E_2_; PI3K/AKT: phosphatidylinositol 3-kinase/protein kinase B; PKC: protein kinase C; PON2: paraoxonase 2; PPAR-γ: peroxisome proliferator-activated receptor gamma; PPMS: primary progressive MS; PRRs: pattern recognition receptors; PUFAs: polyunsaturated fatty acids; RANTES: regulated upon activation, normal T-cell expressed and secreted; ROS: reactive oxygen species; RRMS: relapsing-remitting multiple sclerosis; PYD: pyrin domain; SCI: spinal cord injury; SFAs: saturated fatty acids; SGLT2i: sodium-glucose cotransporter-2 inhibitor; sIL-6R: soluble IL-6 receptor; SNPs: single nucleotide polymorphisms; SIRT1: sirtuin 1; SOCS1/3: suppressor of cytokine signaling 1 and 3; SOD: superoxide dismutase; STAT1: signal transducer and activator of transcription 1; STAT3: signal transducer and activator of transcription 3; SPMS: secondary progressive MS; T2DM: type 2 diabetes mellitus; Th1: T helper 1 cell; Th17: T helper 17 cell; TLRs: Toll-like receptors; TLR-2: Toll-like receptor 2; TLR-4: toll-like receptor 4; TLR-8: Toll-like receptor 9; TNF-α: tumor necrosis factor-alpha; TNFR1: tumor necrosis factor receptor 1; tPAs: tissue plasminogen activators; Treg: regulatory T cell; TSLP: thymic stromal lymphopoietin; TYK2: tyrosine kinase 2; TXNIP: thioredoxin-interacting protein; ULK1: UNC-51-like kinase; VCAM-1: vascular cell adhesion molecule 1; WHO: World Health Organization; Zn^2+^: zinc cation.
